# Performance of Acacia Gum as a Novel Additive in Thin Film Composite Polyamide RO Membranes

**DOI:** 10.3390/membranes9020030

**Published:** 2019-02-15

**Authors:** Yehia Manawi, Viktor Kochkodan, Ahmad Fauzi Ismail, Abdul Wahab Mohammad, Muataz Ali Atieh

**Affiliations:** 1 Qatar Environment and Energy Research Institute (QEERI), Hamad bin Khalifa University (HBKU), Qatar Foundation, 34110 Doha, Qatar; yehmanawi@hbku.edu.qa; 2 Advanced Membrane Technology Research Centre (AMTEC), Universiti Teknologi Malaysia, 81310 Skudai, Johor, Malaysia; afauzi@utm.my; 3 Department of Chemical and Process Engineering, Faculty of Engineering and Built Environment, Universiti Kebangsaan Malaysia, 43600 Bangi, Selangor Darul Ehsan, Malaysia; drawm@ukm.edu.my; 4 College of Science and Engineering, Hamad Bin Khalifa University, Qatar Foundation, PO Box 5825, 34110 Doha, Qatar

**Keywords:** reverse osmosis, polyamide membrane, salt rejection, Acacia gum, interfacial polymerization, hydrophilicity, surface charge, antifouling properties

## Abstract

Novel thin film composite (TFC) polyamide (PA) membranes blended with 0.01–0.2 wt.% of Acacia gum (AG) have been prepared using the interfacial polymerization technique. The properties of the prepared membranes were evaluated using contact angle, zeta potential measurements, Raman spectroscopy, scanning electron microscopy, and surface profilometer. It was found that the use of AG as an additive to TFC PA membranes increased the membrane’s hydrophilicity (by 45%), surface charge (by 16%) as well as water flux (by 1.2-fold) compared with plain PA membrane. In addition, the prepared PA/AG membranes possessed reduced surface roughness (by 63%) and improved antifouling behavior while maintaining NaCl rejection above 96%. The TFC PA/AG membranes were tested with seawater collected from the Arabian Gulf and showed higher salt rejection and lower flux decline during filtration when compared to commercial membranes (GE Osmonics and Dow SW30HR). These findings indicate that AG can be used as an efficient additive to enhance the properties of TFC PA membranes.

## 1. Introduction

Membrane desalination by reverse osmosis (RO) is the most-frequently used technology to provide freshwater from saline water in industrial scale. RO polyamide (PA) membranes are the most commonly used membranes which are prepared via interfacial polymerization (IP) between two monomers, and the polymerization reaction occurs in the interface between the organic and aqueous phases [1,2]. Trimesoyl chloride (TMC) in n-hexane and m-phenylenediamine (MPD) in water are the two phases, which are the most commercially used in the fabrication of thin film composite (TFC) membranes. In such membranes, the skin layer (PA layer) is anchored on top of a porous substrate layer by “in situ polycondensation process” [2,3].

The introduction of different additives to the TFC membrane during the preparation process has been widely reported in the literature to adjust some important membrane properties such as: surface charge, roughness, hydrophilicity, fouling resistance, and chemical stability [4,5,6,7,8,9,10,11,12,13,14]. For instance, the PA thin film nanocomposite (TFN) RO membrane (doped with zeolite nanoparticles) prepared by Jeong et al. [15] demonstrated smoother, more negatively-charged and more hydrophilic surfaces along with the increase in the zeolite loading. This resulted in enhancing the permeability by more than 80% at the highest loading of zeolite. Similarly, Barona et al. [2] and Amini et al. [16] prepared two TFN PA membranes incorporated with aluminosilicate single-walled carbon nanotubes (SWNTs) and functionalized multi-walled carbon nanotubes (MWCNTs) and found the membrane’s hydrophilicity and permeate flux to increase by 1.5-fold and 160%, respectively. Elimelech et al. [17] studied the effect of adding the anionic surfactant sodium dodecylsulphate to a TFC RO membrane on surface morphology and fouling rate of the prepared membrane. It was observed that the introduction of the surfactant resulted in the smoothening of the PA TFC membrane and a reduction of the membrane fouling. They reported that the rougher the membrane surface is, the larger the skin surface area and the higher the rate of colloidal attachment onto the membrane surface are. This results in a higher fouling rate and hence, lower permeates flux with time [4,18,19].

Tarboush et al. [4] used hydrophilic surface-modifying macromolecules (SMM) as an additive for the preparation of a TFC RO membrane by IP technique on porous polysulfone support. It was reported that SMM were incorporated into the aromatic PA layer of the TFC membrane effectively and the prepared membranes exhibited less flux decay over an extended operational period due to a change in the membrane’s hydrophilicity and surface roughness.

Rana et al. [20] added SMM containing polyethylene glycol to the casting solutions to increase the hydrophilicity of the polymeric membranes prepared by phase inversion technique. The modified membranes showed higher hydrophilicity and higher fouling resistance when compared to pristine membranes [4,20,21,22,23,24]. The SMM blended membranes have been reported to demonstrate a higher performance when tested in ultrafiltration and microfiltration applications [20,22,24,25].

**Figure 1:**
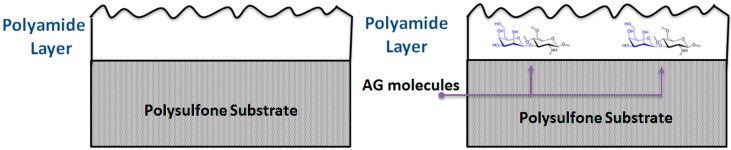
Schematic showing the incorporation of AG inside the top layer of the PA TFC membrane.

In this work, Acacia gum (AG) for the first time was used as an additive during preparation of TFC RO membranes. Acacia gum is a natural gum which is collected as exudation from the stem and branches of Vachellia (Acacia) seyal and Acacia Senegal [26]. AG is a complicated blend of polysaccharides and glycoproteins. The main amino acids present in AG are: histidine, glycine, alanine, arginine, and glutamic acid, whereas the main monosaccharides are: galactose, arobinopyranose, rhamnose, and glucuronic acid [26]. AG is widely used in industry as an edible surfactant, emulsifier and stabilizer (E414) in addition to numerous other applications in various fields. The reason behind the wide use of AG compared to other natural gums is due to its good emulsifying properties, high solubility, low viscosity, and non-toxicity [27]. The amphiphilic nature of AG has been reported to occur due to the simultaneous existence of hydrophilic polysaccharides fragments and hydrophobic protein chains in AG macromolecules [28]. This amphiphilic behavior is believed to be responsible for emulsifying properties of AG when hydrophobic residues adsorb to the oil droplet surface, whereas the hydrophilic ramification limits the droplet aggregation and coalescence via the steric and/or repulsive electrostatic forces [28,29,30,31,32]. According to Gashua et al. [33], AG has been widely used in industry to stabilize the flavor oil in water emulsions [34]. Acacia gum was used as an additive to enhance the properties of PES UF membranes prepared a by phase inversion process by incorporating AG into the dope solution [35]. The prepared membranes possessed higher hydrophilicity, surface charge, and smother surface roughness. In that previous work, AG was incorporated through the whole porous matrix of a PES membrane. In this work, for the first time, AG was incorporated to the active (top) layer of the PA TFC membrane as shown in Figure 1. The effect of the addition of AG on the hydrophilicity, surface roughness, flux, salt rejection, chlorine, and fouling resistance of the prepared TFC PA/AG membranes was discussed. It was shown that the amphiphilic nature of AG can be utilized to enhance the performance of PA TFC membranes in terms of flux and antifouling properties.

## 2. Materials and Methods

### 2.1. Materials

1,3 phenylenediamine (MPD) and ssodium hypochlorite were purchased from Merck (Kenilworth, NJ, USA). 1,3,5-Benzentricarboxylic acid chloride (TMC) was acquired from Acros (Kenilworth, NJ, USA). N-hexane and sodium alginate were purchased from Sigma Aldrich (St. Louis, MO, USA). Polysulfone ultrafiltration membrane (PS-20 UF) from SEPRO Membranes (Carlsbad, CA, USA) with a molecular weight cut-off of 20 kDa was used as a commercial substrate for the preparation of TFC membranes. For comparitive purposes, two commercial RO PA TFC membranes from GE Osmonics (Delfgauw, The Netherlands) and Dow SW30HR were used in this work.

### 2.2. TFC Membrane Fabrication

Several loadings of AG, namely: 0.01, 0.03, 0.05, 0.07, 0.10, and 0.20 wt.% were added to the TMC/n-hexane solution and stirred overnight. The thin PA layer was introduced on top of the commercial substrate via IP technique. The fabrication of the membrane was conducted as follows: First, the commercial substrate was soaked in water for 48 h to remove air bubbles. Then, the excess water from the surface of the substrate was wiped off using a rubber roller. Forty milliliters of MPD solution (2 wt.% of MPD in deionized water) was poured on top of the substrate and the solution was kept for 2 min. The MPD solution was then decanted and wiped off using a rubber roller. After that, 0.1 wt.% solution of TMC in n-hexane was poured on the substrate surface and kept for 1 min to allow the polymerization process to take place. The TMC solution was then decanted and wiped off using a rubber roller. The membrane was left to dry for 1 min before inserting it inside an oven at 60 °C for 5 min. Finally, the membrane was taken out and soaked in deionized water overnight before testing.

### 2.3. Membrane Characterization and Testing

#### 2.3.1. Surface Morphology and Porous Structure

In order to analyze the morphology of the membranes, the top and cross-section views of the fabricated membranes were observed using Field Emission Scanning Electron Microscopy (FESEM) (Gemini model SUPRA 55VP-ZEISS, Carl Zeiss, Dresden, Germany). Liquid nitrogen was used to break the membrane samples and platinum was used to coat the top of the membrane’s surfaces prior to SEM scanning.

#### 2.3.2. Membrane Surface Charge and Hydrophilicity

SurPass 3 electrokinetic analyser (Anton Paar KG, Graz, Austria) was utilized to figure out the zeta potentials of the membranes. The Helmholz–Smoluchowsky equation was implemented to estimate the zeta potential value on the membrane’s surface from the slope of the streaming potential versus the operating pressure curve:

(1) $$\zeta =\frac{\Delta E\mu k}{\Delta P\varphi_{0}\varphi_{r}}$$

where, Δ*P* is the pressure drop across the membrane, $\varphi_{0}$ is the vacuum permittivity, *μ* is the solution viscosity, $\varphi_{r}$ is the dielectric constant of water (at 25 °C), *k* is the conductivity of the electrolyte, and Δ*E* is the streaming potential. In this work, the zeta potential of the membrane surfaces was measured at different pH values (acidic, neutral and basic conditions) by changing the pH of the electrolyte solution using 0.1 M HCl and 0.1 M NaOH solutions.

The hydrophilicity of the membranes was estimated by measuring the contact angle of the water droplet of 2.0 μL with the membrane surfaces using a Ramé-hart standard contact angle goniometer (USA).

#### 2.3.3. Fourier Transform Infrared (FTIR)

FTIR spectra of the membrane samples and the permeate collected were recorded using a Nicolet 6700 Thermo Scientific-FITR spectrometer (Thermo Scientific, Waltham, MA, USA).

#### 2.3.4. Membrane Filtration Tests

The filtration tests were carried out using a 300-mL Sterlitech (Sterlitech, Kent, WA, USA) dead-end filtration cell (HP4750X), which has a membrane cross-section area of 14.6 × 10^−4^ m^2^. The liquid in the cell was pressurized using nitrogen gas, and permeate flux was calculated from Equation (2):

(2) $$J=\frac{V}{A\cdot t}$$

where *V* is the permeate volume (L), *A* is the membrane cross-sectional area (m^2^), and *t* is the permeation time (h).

The salt rejection tests were conducted with 2000 ppm NaCl solutions at pH 6–7 and at operating pressure of 15 bars. Additionally, the filtration tests with real (untreated) seawater at pH 8.36 and at operating pressure of 54 bars were carried out. The seawater filtration experiments were carried out on 2 consecutive days (48 h). About 280 mL of seawater was added at the beginning of every day and the filtration was conducted. The degree of permeate recovery was 60%. At the end of the filtration run, the membrane was washed with DW for 15 min before starting another filtration run.

Equation (3) was used to figure out the salt rejection (in %):

(3) $$R\left(\%\right)=\left(1-\frac{C_{p}}{C_{f}}\right)\times 100\%$$

where *C_p_* and *C_f_* (in ppm) stand for the salt ions concentration in the permeate and feed solutions, respectively. NaCl concentration was determined by measuring the electrical conductivity of the feed and permeate solutions using Thermo Scientific Orion Conductivity Benchtop Meter (Thermo Scientific, Waltham, MA, USA) after appropriate calibration. The trace metals’ concentration in the feed, brine and permeate were measured using a Thermo Fisher iCAP 6500 Duo—Inductively Coupled Atomic Emission Spectrometer (ICP-AES, Thermo Scientific, Waltham, MA, USA) instrument. Moreover, Thermo Scientific Dionex ICS-5000+ Capillary HPIC (Thermo Scientific, Waltham, MA, USA) was used to figure out the cations and anions concentration in the probes.

The antifouling performance of the prepared membranes was studied by evaluating the normalized flux of the membranes after the filtration of 100 ppm sodium alginate solution at an operating pressure of 15 bars. In this test, the pure water flux of the membranes (*J_i_*) was first determined for 15 min. After that, 100 ppm sodium alginate solution was filtered through the membranes for 2 h. In the end, the membrane was rinsed with deionized water (DW) and pure water flux (*J_f_*) for 15 min was measured again. The normalized flux (*J_n_*) was then calculated using Equation (4):

(4) $$J_{n}=\frac{J_{f}}{J_{i}}$$

#### 2.3.5. Surface Morphology and Roughness

The surface morphology of the membrane’s surfaces were analyzed using KLA Tencor P-17 Stylus Profiler (Tencor, Milpitas, CA, USA). This profiler has a Stylus probe diameter of 2 µm and 200 mm scan length in X-Y and Z-resolution of 10 Angstrom.

#### 2.3.6. Membrane Chlorine Stability Tests

In order to account for the chlorine resistance test of the prepared membranes, sodium hypochlorite solution (1000 ppm) was used. In this test, the salt rejection and water permeability were assessed for the PA/AG membranes and AD commercial membrane before immersion in NaOCl solution. After that, the membranes were washed and immersed in the sodium hypochlorite solution for 24 h. The membranes were taken out of the NaOCl solution, washed sufficiently with DW water before testing their permeability and salt rejection using the same procedure described above. The change in the permeability and salt rejection was then determined and analyzed.

### 2.4. Seawater Sampling

**Figure 2:**
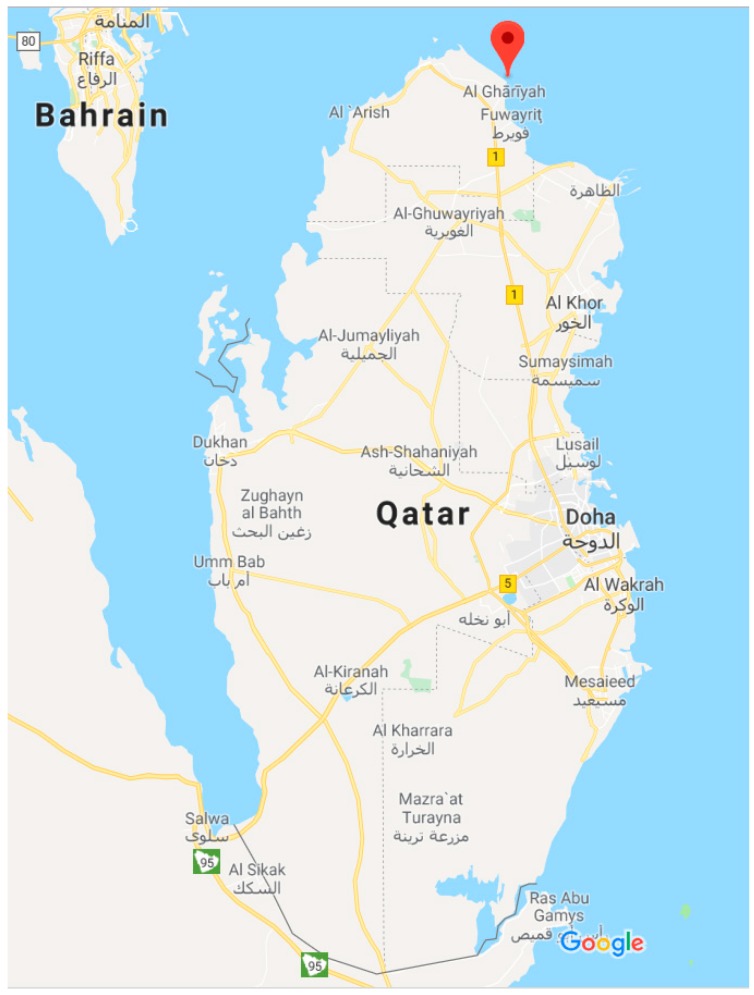
Map showing the location of the seawater sampling near the Arabian Gulf.

Seawater samples were collected from the Arabian Gulf about 200 meters away from the coast line in order to reduce the influence of anthropogenic contaminants. As shown in Figure 2, the samples were obtained from the northen part of Qatar near the Al-Ghariyah beach. The physico-chemical parameters of the collected seawater samples are presented in Table 1. The seawater was collected using amber glass and polyethylene containers. In order to analyze the inorganic content, the seawater sample was acidified with 2 wt.% HNO_3_.

## 3. Results

### 3.1. Membrane Morphology and Hydrophilicity

**Figure 3:**
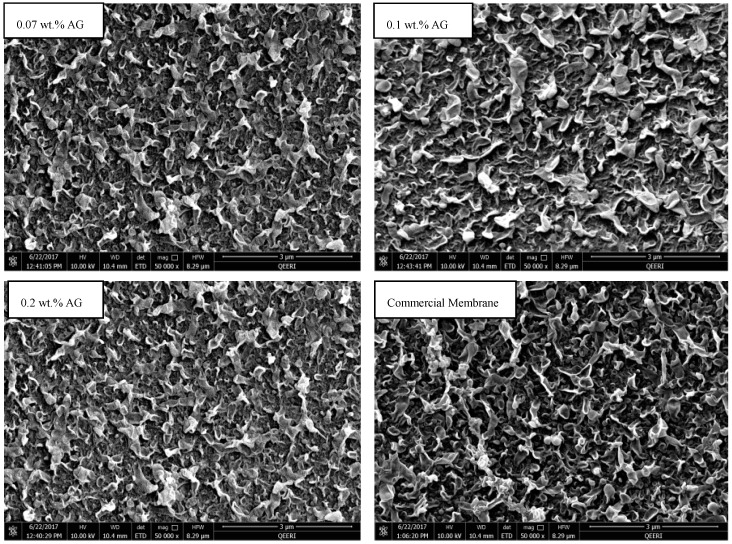
SEM top view of PA membranes with 0.07, 0.1 and 0.2 wt.% AG in dope solution in addition to the commercial AD Osmonic membrane.

**Figure 4:**
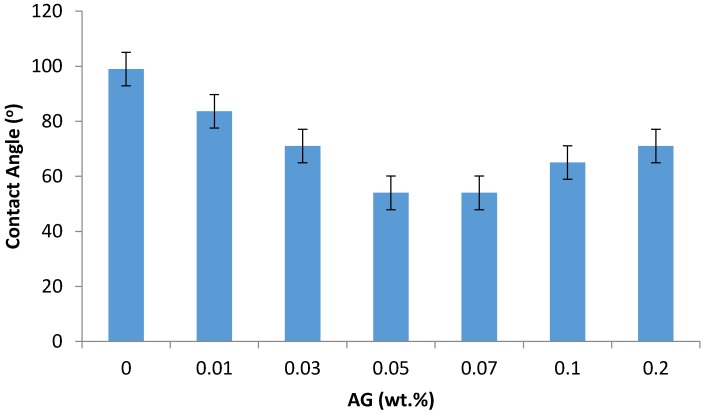
The contact angle of the prepared PA/AG membranes at different AG (wt.%) loadings in dope solutions.

The top surface and cross section of the fabricated membranes were inspected using FE-SEM (Carl Zeiss, Dresden, Germany). Figure 3 depicts the top views of PA/AG-containing membranes with different AG loading in addition to the commercial PA RO membrane. On the other hand, Figure 4 shows the cross-section of the PA membrane without AG and PA/0.1 wt.% AG membranes. As seen in Figure 3, the membrane’s top surfaces were uniform and there were no defects in their morphology. The surface topography of the fabricated membranes was similar to that of the commercial AD Osmonic membrane. Furthermore, the cross-section SEM images show practically no difference between the fabricated bare and AG-containing membranes.

As seen in Figure 4, adding AG to PA membranes increases the membrane hydrophilicity by reducing the contact angle by up to 45% (at 0.07 wt.% AG) when compared to bare PA membrane. This hydrophilization effect is believed due to the amphiphilic nature of the AG macromolecules, which include both hydrophilic polysaccharides fragments and hydrophobic protein chains [28]. When introducing AG into the IP process, obviously the hydrophobic fractions of the AG macromolecules bind with the hydrophobic PA backbone, while leaving the hydrophilic AG residues (carbohydrates) to hydrophilize the membrane surface.

The drop in water contact angle (and hence increase in the hydrophilicity) has been found to reduce the fouling by the formation of hydrogen bonds between the water molecules and membrane surface, minimizing the interaction between the hydrophobic foulants and the membrane surface [36]. Some increase in the PA/AG membrane hydrophobicity at AG loading beyond 0.07 wt.% can be attributed to the possible aggregation of AG molecules in TMC solution, which tends to reduce the hydrophilization effect at high AG loading.

In this work, the addition of AG to PA TFC membranes was found to be more efficient in enhancing the hydrophilicity than the addition of some other nanomaterials reported in literature. Rajaeian et al. [37] fabricated a TFN membrane by incorporating aminosilanized TiO_2_ nanoparticles to a PA membrane. The contact angle of the optimized membrane surface was found to be 75.8°. Likewise, Sorribas et al. [38] fabricated another TFN membrane through the addition of aluminum and chromium organic frameworks nanoparticles. They found that the addition of the nanoparticles resulted in the reduction of the contact angle down to 50°.

### 3.2. DSC Thermograms

**Figure 5:**
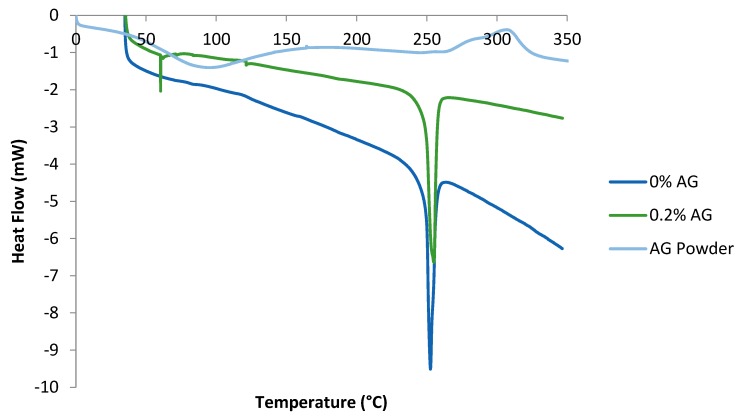
Thermograms of plain PA membrane (0% AG), PA/0.2 wt.% AG membrane and AG powder.

The DSC of pure AG in addition to that of PA/AG membranes is shown in Figure 5. In the AG thermogram, the endothermic peak shown at 90 °C signified the loss of water absorbed by AG in the form of moisture, whereas the exothermic peaks shown at 300 °C indicated the decomposition of AG. These peaks were found to agree with the DSC study conducted by other researchers who analyzed four AG samples from different Acacia species [27]. Their temperature ranges were found to lie between 100–150 °C for the endothermic peaks and 300–315 °C for the exothermic peaks. Figure 5 also depicts the DSC of PA/AG membranes containing varying loadings of AG. As shown, the presence of the endothermic peaks is clearly shown at around 250 °C. These endothermic peaks represent the glass transition temperatures (T_g_) of the PA/AG membranes and it is seen that AG addition has no effect on the T_g_ of TFC PA/AG membranes. Moreover, the presence of the small endothermic peak at about 55 °C corresponded to the loss of water which exists as a result of the presence of AG inside the membrane.

### 3.3. Membrane Surface Charge

The effect of AG on the membrane’s surface charge was studied by measuring the zeta potential of the membrane samples at different pH’s of the feed solution. As seen in Table 2, the zeta potential of the PA/AG membranes became negative with the increase in the pH values of the solution, which is obviously due to deprotonation of amino groups and dissociation of carboxylic groups in incorporated AG macromolecules. At pH values higher than 1.9, Naiu et al. [39] reported that the macromolecules of AG behave as a weak polyelectrolyte carrying a negative surface charge; they explained this behavior to occur due to the dissociation of carboxyl groups of AG.

The zeta potential values of the bare PA membrane in this work were found to agree with those reported in the literature at the same pH values (*ζ* = 16 and −43 at pH = 3 and 8.5, respectively) [40]. The increase in the negative zeta potential values of the PA membranes along with the increase in the pH was studied and reported by several researchers and is thought to occur due to the deprotonation of the functional groups on the membrane surface [41,42,43].

As seen in Table 2, in acidic conditions, the membranes demonstrated positive zeta potential values. This might be explained by the protonation of the functional R-C=O-NH-R groups in the PA membrane as well as amino groups of AG at these conditions. Interestingly, the AG-containing membrane showed relatively lower positive zeta potential values when compared with pure PA membrane at pH 3.2. The reason behind this decrease is not clear yet as the presence of amino groups in AG macromolecules, which can be protonated at these conditions, is expected to result in higher positive zeta potential of PA/AG membranes.

### 3.4. Surface Roughness

**Figure 6:**
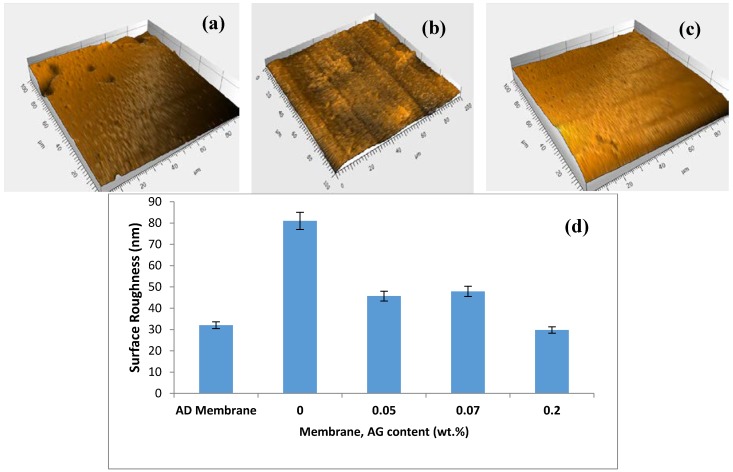
3D images depicting the membrane surfaces: AD commercial membrane (Osmonics) (**a**), neat PA membrane (**b**), PA/0.1 wt.% AG (**c**) and average surface roughness of the studied membranes (**d**). Scanned area 100 × 100 µm^2^.

The surface topography of the prepared PA/AG membranes was studied by using the surface profiler. Figure 6a shows 3D images depicting the top surface of AD commercial membrane (GE Osmonics) and 0.1 wt.% AG membrane. In general, PA/AG-containing membranes showed relatively smoother surfaces (less average surface roughness) when compared with the bare PA membrane (Figure 6b), which is obviously due to the distribution of AG molecules in smoothing the membrane surface and reducing the ridge-valley structure encountered in the interfacially polymarized aromatic PA TFC membranes. Moreover, PA/AG membranes with 0.1 and 0.2 wt.% of AG exhibited lower surface roughness (17.1 and 29.8 nm, respectively) compared to the commercial AD membrane, which had an average surface roughness of about 32 nm. Elimelech et al. [17] reported similar behaviour by the introduction of anionic surfactant during synthesis of PA RO membranes. They found that the introduction of the surfactant resulted in the smoothening of the PA TFC membrane. It should be noted that the values of the surface roughness for PA/AG membranes were lower than that reported in the literature when some nanomaterials were used to fabricate TFN membranes. For instance, Barona et al. [2] and Amini et al. [16] prepared two TFN PA membranes embedded with aluminosilicate-functionalized single-walled carbon nanotubes and amine-functionalized multi-walled carbon nanotubes and found the average roughness values to be 50.8 and 97.2 nm, respectively. Similarly, PA-TFN membranes impregnated with TiO_2_ and zeolite nanoparticles had an average surface roughness of about 79.2 nm [37] and 57 nm [15], respectively.

It was postulated that the reducing of surface roughness of PA/AG membranes might be attributed to the increase in the miscibility between the organic and aqueous phases in IP process, when hydrophilic AG is added, that results in smoothening of the ridge-valley structure of the surface of the prepared PA/AG membranes. A similar trend was reported by Mahboub et al. [44] who found that the addition of the hydrophilic amino-functionalized UZM-5 nanoparticles during the IP increased the miscibility of the aqueous and organic phases and that resulted in the transformation of the ridge and valley surface membrane morphology.

### 3.5. Filtration Tests

**Figure 7:**
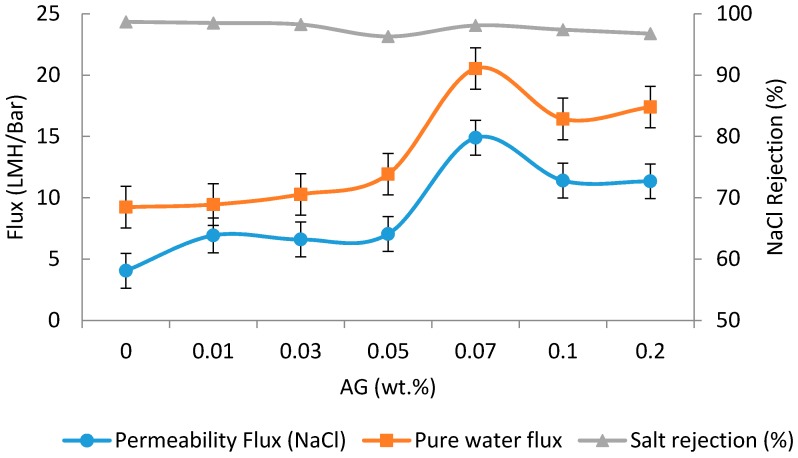
Fluxes and salt rejection of PA/AG-containing membranes at different AG (wt.%) loadings in dope solutions. Feed solution: 2000 ppm NaCl, pH 7. Operating pressure: 15 bars.

The water fluxes and NaCl rejection values with PA/AG membranes at different AG loadings are depicted in Figure 7. As shown, the addition of AG to TFC membranes has the effect of increasing the pure water flux and flux during filtration of the NaCl solution by 1.2- and 2.6-fold, respectively at 0.07 wt.% AG. Interestingly, the increase in flux of the TFC membrane did not compromise the salt rejection significantly (Figure 7). The effect of AG loading to PA membrane was found to increase the flux initially and then decrease (beyond 0.07 wt.% AG loading in dope solution). The increase in the membrane’s flux can be attributed to the improvement in the PA/AG membrane hydrophilicity. Another explanation for this behavior can be attributed to the enhancement in the miscibility between the organic and aqueous phases when hydrophilic AG is added, which resulted in the formation of a less cross-linked PA layer. Similar behavior was also reported by other researchers, who attributed the enhancement in the miscibility between the organic and aqueous phases to the increase in the additives loading to TFN membranes [44,45]. Moreover, as reported by Mahboub et al. [44] and Ghosh et al. [46], the increase in the loading of zeolite nanoparticles resulted in the increase of the viscosity of the TMC solution and hence, reduces the diffusivity of the MPD in the organic phase. These two factors had the effect of producing a less cross-linked PA membrane surface [44,47].

On the other hand, the decrease in the membrane flux at AG loading above 0.07 wt.% is thought to occur due to the decrease of membrane hydrophilicity at high AG loading due to possible AG aggregation. It should be noted that the use of AG as an additive resulted in a significant increase in the PA/AG membrane flux while keeping NaCl rejection above 96%. This increase in flux has been found to be comparable to that observed when TFN membranes were incorporated with zeolite, TiO_2_, SiO_2_, aluminosilicate nanoparticles, and CNTs [2,15,37,48,49].

### 3.6. Fouling Tests

**Figure 8:**
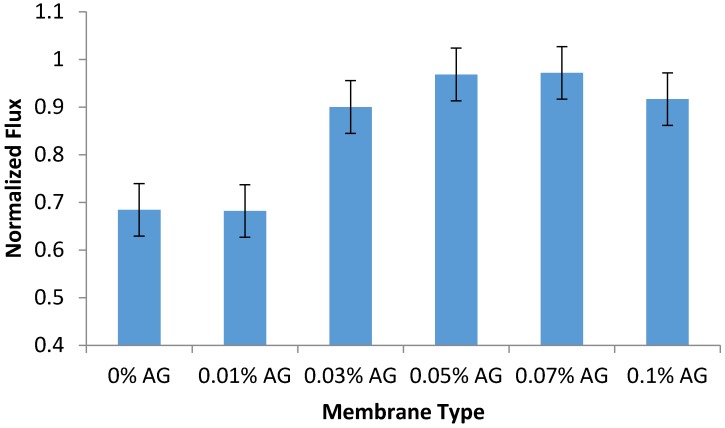
Normalized flux of PA/AG membranes (at different AG loadings in dope) after filtration of 100 ppm sodium alginate solution at an operating pressure of 15 bars for 2 h. pH: 6.95.

The antifouling performance of the prepared PA/AG membranes was evaluated by calculating the normalized flux of the membrane samples after the filtration of 100 ppm sodium alginate solution as described in the methodology section. As seen in Figure 8, the addition of AG to PA membranes was found to increase their normalized flux and antifouling properties (by 44%) when compared to the bare PA membrane. This can be attributed due to the increase in the hydrophilicity, surface charge and drop in surface roughness of PA/AG membranes, which manifested itself in the reduction of membrane fouling. The surface charge, roughness and hydrophilicity of the membranes have been widely reported to influence the membrane flux, rejection, and fouling resistance [4,18,19].

### 3.7. Stability of PA/AG Membranes

#### 3.7.1. FTIR of the Permeate Samples

**Figure 9:**
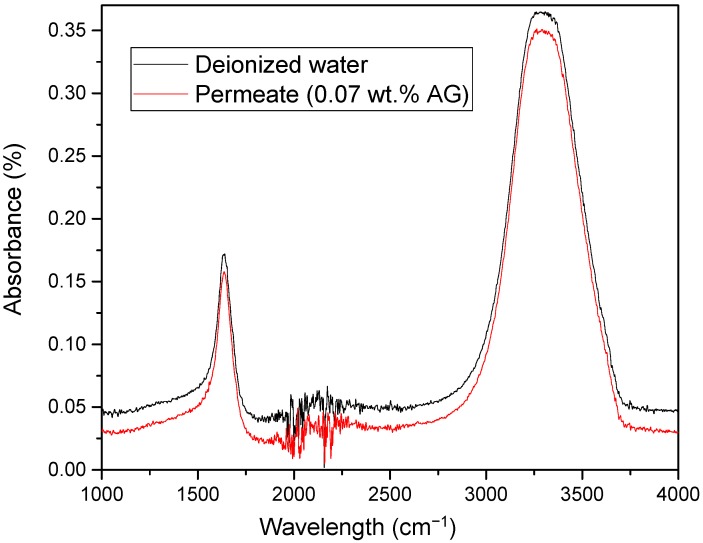
FTIR spectra of the permeate with PA/0.07 wt.% AG membrane in comparison with the spectra of DI water.

The stability of incorporation of AG in PA/AG membranes was tested by analyzing the FTIR spectra of the permeate samples collected during the filtration of DI water. Figure 9 shows a FTIR spectra of the permeate with PA/0.07 wt.% AG membrane in comparison with the spectra of the DI water. As depicted, the spectra of the permeate with the PA/AG membrane was identical to that of the DI water. This indicates that there is no leaching of AG from PA/AG membrane.

#### 3.7.2. Chlorine Resistance

**Figure 10:**
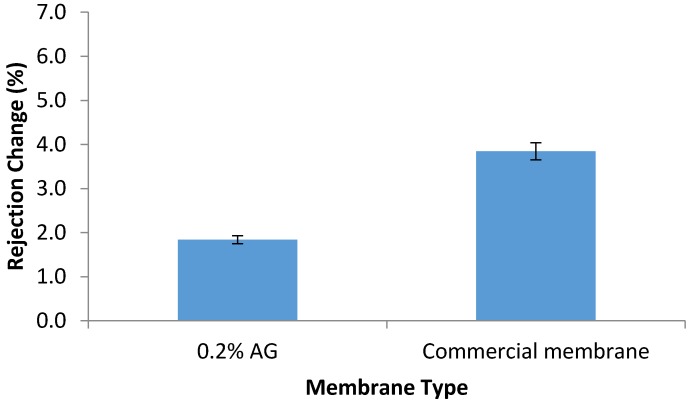
Change in the salt rejection of the AD commercial membrane and PA/0.2 wt.% AG membrane after immersion in 1000 ppm of NaOCl solution for 24 h.

The chlorine resistance test was conducted by the immersion of the prepared PA/AG membranes in 1000 ppm NaOCl solution for 24 h. As seen in Figure 10, rejection capability of PA/0.2 wt.% AG membrane was less affected by the hypochlorite when compared with the AD commercial membrane. The higher resistance of the PA/AG membrane to hypochlorite can be attributed to the shielding effect of the AG macromolecules that reduce amide bond cleavage of the PA polymer network.

Sodium hypochlorite dissociates in aqueous solutions to form sodium cation and hypochlorite anion, which is a strong oxidizing agent:

NaOCl(aq) ⇌ Na^+^(aq) + OCl^−^(aq)

It was reported that the number of carboxylic groups on the PA surface, which appear to form by hydrolysis of the amide bonds (C(O)-N), increased after contact of the PA membrane with hypochlorite solution [50]. Since AG includes both polysaccharides residues and the protein chains with numerous amide bonds [26,33], some hypochlorite ions will be consumed for hydrolysis of amide linkages in AG macromolecules, and this will reduce the degradation of the PA backbone of the PA/AG membrane.

It is also possible that the introduction of high AG loading (0.2 wt.%) to TMC solution alters the thermodynamic balance in the organic phase and this might result in the formation of a more cross-linked top PA layer with better chemical stability. The improved chlorine resistance of PA/AG membranes might extend the membrane lifetime as feed water chlorination is often used to reduce membrane bio-fouling.

### 3.8. Membrane Performance with Seawater

Table 3 lists the composition of the feed seawater, brine and permeate from TFC PA/0.2 wt.% AG membrane. As seen in the table, the permeate from the PA/AG membrane showed substantially lower TDS, salt ions and metals content when compared with the feed seawater.

**Figure 11:**
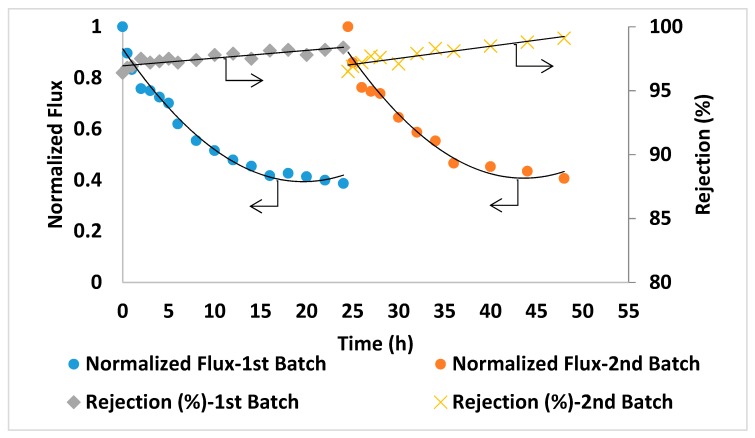
Normalized flux and salt rejection vs time of PA/AG membrane at 0.2 wt.% AG in the dope solution. TDS of the feed solution: 45,000 ppm, pH 8.36. Operating pressure: 54 bars.

The prepared PA/AG membranes were tested by carrying out a filtration experiment using Qatari seawater without any pre-treatment. The filtration experiment was carried out continuously for 48 h. The initial permeate flux was found to be reasonably high (above 5 LMH) for this high salinity feed solution. The salt rejection was found to slightly increase and reach up to 99.1% with filtration time (Figure 11). This might be attributed to some compaction of the membrane porous structure as well as the formation of a cake/gel layer from suspended, organic and microbiological matter in seawater on the membrane surface that resulted in a higher salt rejection [51]. Figure 11 shows the reduction in the normalized permeate flux with time (from 1 at the beginning of the time down to 0.3 after 24 h). This might be explained by an increase in salt concentration and in turn, in the osmotic pressure of the feed seawater in the membrane cell during filtration. It is worth-mentioning that the TDS of the brine solution after 24 h of filtration time has increased by more than 55% (from 45,000 ppm at the beginning of the experiment up to about 70,000 ppm at the end of each batch). For comparison, PA membranes incorporated with aluminosilicate SWNTs [2] showed 96% salt rejection with 585 ppm NaCl solution [2] and 88–92.4% rejection with 2000 ppm NaCl solution was reported for PA MWCNTs membranes [16]. As seen from this comparison, PA/AG membranes showed much higher rejection when testing with real seawater (about 45,000 ppm), which is an indication of the enhancement in the performance of the AG-containing membranes.

**Figure 12:**
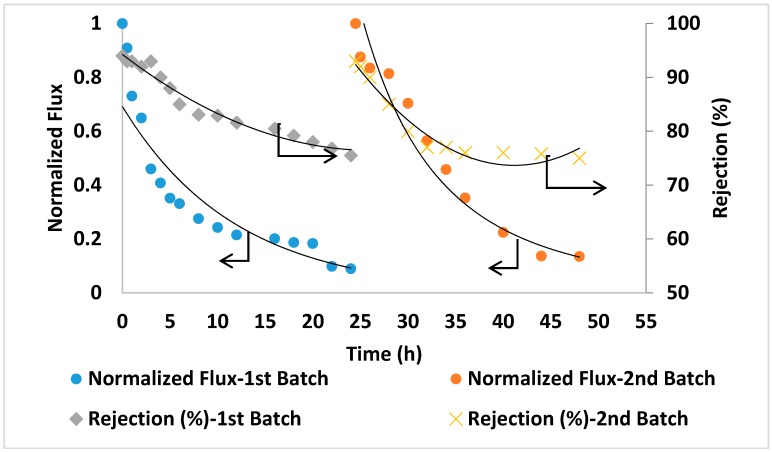
Flux and salt rejection vs time of the commercial membrane (Osmonics). TDS of the feed solution: 45,000 ppm, pH 8.36. Operating pressure: 54 bars.

**Figure 13:**
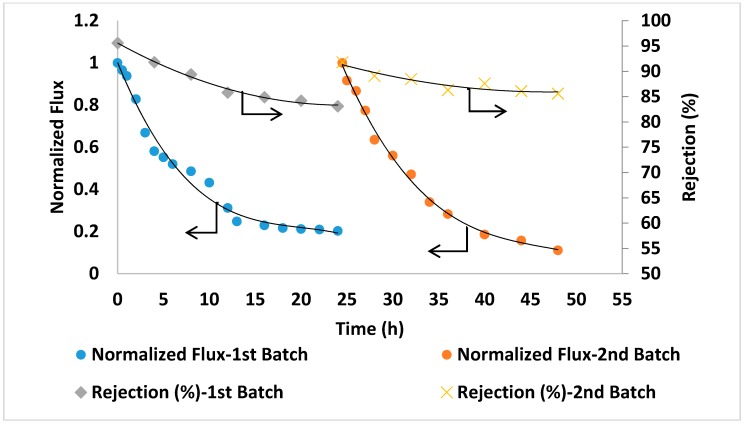
Flux and salt rejection vs time of the commercial membrane (Dow SW30HR). TDS of the feed solution: 45,000 ppm, pH 8.36. Operating pressure: 54 bars.

For comparison in the same conditions, seawater filtration experiments were conducted using two commercial TFC membranes (GE Osmonics and Dow SW30HR) (Delfgauw, The Netherlands and Midland, MI, USA). As shown in Figure 12 and Figure 13, the commercial membranes showed lower overall salt rejection compared to PA/0.2 wt.% AG membranes. Moreover, as shown in Figure 11, Figure 12 and Figure 13, the PA/AG membrane demonstrated higher normalized flux values when compared with commercial membranes. For instance, the normalized flux values after 24 and 48 h filtration time were 0.38 and 0.4 for the PA/AG membrane, while 0.09 and 0.13 for Osmonics and 0.2 and 0.11 for Dow membranes. Lower flux decline for PA/AG membrane might be explained by the enhancement in the membrane properties such as hydrophilicity, negative surface charge and surface roughness.

## 4. Conclusions

Novel TFC PA membranes blended with 0.01–0.2 wt.% of AG have been prepared by IP technique. It was found that the hydrophilicity of PA/AG membranes increased (by up to 45%) compared with the bare PA membrane. This might be due to the amphiphilic nature of AG when hydrophobic parts of AG macromolecules are adsorbing to the hydrophobic PA polymer network while the hydrophilic residues of AG are protruding towards the aqueous phase. In addition, it was shown that PA/AG membranes reduced surface roughness (by 63%) and increased chlorine resistance (by 52%) compared with bare PA membrane. The presence of carboxylic and amino groups in AG macromolecules has been found to increase the negative surface charge of the membrane surface. The membrane flux was also improved with PA/AG membranes as a result of the enhancement in the membrane hydrophilicity and surface charge while maintaining NaCl rejection above 96%. Due to the increase in hydrophilicity and reduction in surface roughness, a significant reduction in the fouling of PA/AG membranes was observed by the increase in the normalized flux (by 44%) when sodium alginate solution was filtered through the membrane. The RO PA/AG membranes were tested with seawater collected from the Arabian Gulf and showed higher salt rejection and lower flux decline during filtration when compared to commercial membranes (GE Osmonics and Dow SW30HR). These findings indicate that AG incorporation into a PA layer can be used to enhance the properties and performance of TFC PA membranes.

## Figures and Tables

**Table 1:** The physico-chemical parameters of the seawater sample collected from Al-Ghariyah beach.

Element	Feed Seawater
X-coordinates	214,548.34
Y-coordinates	482,954.73
Latitude	26.10147
Longitude	51.362099
Temperature (°C)	19
pH	8.36
Turbidity (NTU)	0.29
Electrical Conductivity ($\frac{mS}{cm}$)	63.4
Total Organic Carbon (ppm)	0.52

**Table 2:** Zeta potential of the prepared PA/AG membranes (pure PA and blended with 0.2 wt.% AG in the TMC/n-hexane solution) at different pH values.

pH	Zeta Potential (mV)	
	0 wt.% AG	0.2 wt.% AG
3.2	13.6	9.6
3.7	4.0	2.6
5	−28.6	−24.6
6	−37.4	−41.5
7	−43.3	−50.5
8	−45.5	−51.3
8.5	−45.4	−50.1

**Table 3:** The characterization results of the seawater, brine and permeate from the TFC PA/0.2 wt. % AG membrane.

Element	Feed Seawater	Brine	Permeate
Total Dissolved Solids (ppm)	45,000	70,000	1400
Chlorides (ppm)	27,877	36,273	840
Sulphates (ppm)	3482.08	5286.4	39.03
Sodium (ppm)	14,040.59	17,500	510
Potassium (ppm)	500	700	22
Calcium (ppm)	650	1350	25
**Heavy Metals (ppb)**			
Silicon (Si)	309.85	553.2	23
Antimony (Sb)	15.15	1	0.7
Barium (Ba)	5.4	9	0.3
Strontium (Sr)	5249	7799	148
Boron (B)	30,480	38,060	6880
Molybdenum (Mo)	14,050	23,350	1706
